# The dynamic change of serum S100B levels from day 1 to day 3 is more associated with sepsis-associated encephalopathy

**DOI:** 10.1038/s41598-020-64200-3

**Published:** 2020-05-07

**Authors:** Long Wu, Qing Feng, Mei-Lin Ai, Song-yun Deng, Zhi-Yong Liu, Li Huang, Yu-Hang Ai, Lina Zhang

**Affiliations:** 1 Department of Critical Care Medicine, Xiangya Hospital of Centre-south University, Changsha, 410008 China; 2 National Clinical Research Center for Geriatric Disorders, Xiangya Hospital, Central South University, Changsha, 410008 PR China

**Keywords:** Prognostic markers, Bacterial infection, Disorders of consciousness

## Abstract

We investigated the role of dynamic changes of serum levels S100B protein in brain injury and poor outcome of sepsis. This is a prospective cohort study designed to include 104 adult patients with sepsis who are admitted to ICU from Jan 2015 to Aug 2016. Sepsis was defined as sepsis 3.0. Patients with a GCS score of <15, or at least one positive CAM-ICU score were thought to have brain dysfunction. 59 patients were diagnosed with SAE and the rest 45 patients were diagnosed with non-SAE. Serum S100B was measured on day 1 and 3 after ICU admission. Primary outcomes included brain dysfunction and 28-day/180-day mortality. The SAE group showed a significantly higher APACHE II score, SOFA scores, length of ICU stay, 28-day and 180-day mortality, serum S100B levels on day 1 and day 3. S100B levels on day 1 of 0.226 μg/L were diagnostic for SAE with 80.0% specificity and 66.1% sensitivity, and the area under (AUC) the curve was 0.728, S100B levels on day 3 of 0.144 μg/L were diagnostic for SAE with 84.44% specificity and 69.49% sensitivity, and the AUC was 0.819. In addition, the AUC for S100B on day 3 for predicting 180-day mortality was larger than for S100B on day 1 (0.731 vs. 0.611). Multiple logistic regression analysis showed that S100B3 (p = 0.001) but not S100B1 (p = 0.927) were independently correlated with SAE. Kaplan-Meier survival analysis showed that patients with S100B levels higher than 0.144 μg/L had a lower probability of survival at day 180. There were more patients with encephalopathy and a higher 28-day or 180-day mortality in the ΔS100B + group than in the ΔS100B- group. Multiple logistic regression analysis showed that SAE and IL-6 on day 3 were independently correlated with S100B dynamic increase. These findings suggest that elevated serum S100B levels on day 3 and the dynamic changes of serum S100B levels from day three to one were more associated with brain dysfunction and mortality than that on day 1 in patients with sepsis.

## Introduction

Sepsis has recently been redefined as a syndrome that causes life-threatening multiple organ dysfunction due to disordered host response to an infection^1^. The brain is one of the most frequently injured organs in sepsis, with an incidence rate of over 50%^2,3^. Brain injury in sepsis clinically manifests as delirium, unresponsiveness, confusion, or even coma. There has been an increased mortality rate in sepsis patients who present with brain injury^4^ and a subsequent decrease in their long-term cognitive function and quality of life^5,6^.

Sepsis-associated encephalopathy (SAE) is a non-specific diffuse brain dysfunction in sepsis patients, in the absence of intracranial infections or other known causes of brain dysfunction. A range of pathophysiological mechanisms has been proposed for the occurrence and development of SAE^7–9^. Injury to the blood-brain barrier (BBB) has been indicated to be an important link in the proposed mechanisms^10–13^. The biomarker, S100 beta (S100B) in serum, has been shown to be a credible marker for assessing the severity of brain injury^14,15^ and predicting the outcomes of traumatic brain injury (TBI)^16^, stroke^17^, hypoxic-ischemic encephalopathy^18^, and postoperative delirium^19^. This biomarker could, therefore, reflect glial cell injury and the blood-brain barrier destruction in sepsis. A growing number of studies have suggested that increased serum S100B levels are associated with brain dysfunction in sepsis patients^20–22^. However, other studies have also shown that neuron-specific enolase (NSE) levels but not S100B levels in severe sepsis or septic shock could predict fatal outcome in patients^23,24^. An observational study with a small sample size reported that physicians could not distinguish between patients with different severities of encephalopathy in sepsis based on serum S100B^25^. In contrast to that, our previous work verified that S100B in serum, rather than NSE, was a better biomarker for SAE^26^. However, the sensitivity and specificity of S100B for the diagnosis and prognosis of SAE were not excellent. Serum S100B levels varied rapidly in the first 24–48 hrs after brain injury^16^, which indicated that serum S100B levels may better reflect the brain damage process in sepsis after 48 hrs.

Unlike other types of brain injury such as traumatic brain injury (TBI) and stroke which have a clear time point of onset, it is challenging to determine a specific time point for the occurrence of SAE. Changes that occur in SAE are continuous and dynamic. Therefore, there is a need to assess biomarkers that exhibit varying responses to the severity of brain injury. The dynamic changes of serum S100B level may make it a viable marker for determining disease severity in septic encephalopathy^27^. A study reported that the average serum S100B level increased during the first 48 hrs in the early death group^20^. Another study showed that the magnitude of the decline in serum S100B levels in the brain dysfunction group was significantly less compared with that in the non-brain dysfunction group within 4 days post-admission^28^. A recent study also suggested that the S100B levels in the plasma of patients with SAE were significantly higher than those in the control group from day 1 to day 7^29^. Furthermore, there was no significant decrease in the plasma S100B levels between day 1 and day 3^29^. To date, only a few prospective studies have aimed at assessing the role of dynamic changes in S100B levels in patients with sepsis brain injury.

Therefore, we designed a prospective cohort study to measure the serum S100B levels on days 1 and 3 in sepsis patients after admission to the intensive care unit (ICU). The aim was to evaluate the role of S100B levels at different time points and to evaluate the dynamic changes in S100B levels on prognosis and brain injury in sepsis.

## Materials and Methods

### Study design and enrollment

This was a prospective cohort study designed to include sepsis patients who were admitted to the comprehensive ICU of Xiangya Hospital from January 2015 to August 2016. This study were approved by the Ethics Committee of Xiangya Hospital, Central South University. Informed consent was obtained from patients or legal representatives. The study was conducted according to the principles of the Declaration of Helsinki.

The inclusion criterion was sepsis patients aged ≥ 18 years. The exclusion criteria were as follows: age <18 years, primary brain injury (such as traumatic brain injury, stroke, cardiac arrest, intracranial infection, epilepsy, Alzheimer’s disease, Parkinson disease and meningitis etc.), acute mental deterioration secondary to non-septic metabolic disorders with organ dysfunction (hepatic encephalopathy, pulmonary encephalopathy, severe electrolyte imbalance, severe blood glucose disorders etc.), psychosis, melanoma, pregnancy or nursing state, severe burns, trauma, neurosurgery, and non-survivors in the first 72 hours from sepsis.

### Clinical protocol

Sepsis is defined as a syndrome that causes life-threatening multiple organ dysfunction due to disordered host response to an infection. Multiple organ dysfunction can be represented by an increase in the Sequential (Sepsis-related) Organ Failure Assessment (SOFA) score of 2 points or more. The assessment of the level of consciousness and delirium was performed in all enrolled patients. We evaluated the level of consciousness by the Glasgow Coma Scale (GCS) before sedation. (For patients who had been sedated prior to ICU admission, the assumed GCS scores, that is, the scores measured before any administration of sedative/relaxant drugs were used for analysis. For postoperative patients, the GCS scores measured before surgery were used.) From the time of ICU admission to the time of discharge from ICU, we evaluated the delirium through the Confusion Assessment Method for the ICU (CAM-ICU). The delirium was assessed twice daily by the nurse or physician in charge of the patient.

Information of the patients’ demographic and clinical characteristics were recorded. The Acute Physiology and Chronic Health Evaluation (APACHE) II scores, and SOFA scores based on the information retrieved within the first 24 hours of ICU admission were also recorded. The 180-day follow-up was mainly conducted by telephone. The return visit ranged from the onset until the fifth to seventh months after being discharged from hospital. The EuroQol 5-dimension questionnaire health scale (EQ-5D) was used to assess the long-term quality of life, which was completed by the patients or their representatives.

The term “S100B1” was used to represent the serum S100B level on day 1 and “S100B3” on day 3. The term ΔS100B was used to represent the value of the serum S100B level on day 3 minus the value on day 1. The term ΔS100B + was used for ΔS100B values that were > 0, and ΔS100B- was used for ΔS100B values that were ≤ 0. The term “S100B1 + ” represented S100B levels that were > the optimal cut-off values of serum S100B levels on day 1 for the diagnosis of SAE. The term “S100B1-” represented S100B levels that were ≤ the optimal cut-off values of serum S100B levels on day one for SAE diagnosis. The term “S100B3 + ” represented S100B levels that were > the optimal cut-off values of serum S100B levels on day 3 for SAE diagnosis. Also, “S100B3-” represented S100B levels that were ≤ the optimal cut-off values of serum S100B levels on day 3 for SAE diagnosis. The differences in EQ-5D scores were assessed between the S100B1 + and S100B1- groups and the S100B3 + and S100B3- groups.

### Assessment of brain dysfunction

SAE was defined as cerebral dysfunction in the presence of sepsis and the absence of any of the exclusion criteria. Refer to the criteria of a recent large-scale study^30^, patients with a GCS score <15 or at least one positive features of delirium were thought to have brain dysfunction. The evaluation of delirium in patients with sedation was accomplished by combining the Richmond Agitation Sedation Scale (RASS) and the daily spontaneous awakening trials. Patients were expected to be awake for delirium evaluation within 24 hours, otherwise they will be considered to have brain dysfunction. Patients who were considered by the clinician to have cerebral hemorrhage or cerebral infarction would be confirmed by the computerized tomography (CT) scan or the magnetic resonance imaging (MRI) after being successfully detached from the ventilator.

### Measurement of serum S100B

We use the ethylenediaminetetraacetic acid (EDTA) anticoagulative tubes to collect blood samples from sepsis patients. Serum S100B concentrations were measured according to the method described in our previous publication^26^.

### Statistical analyses

All the statistical analysis of our data were performed using SPSS 19.0 software (SPSS Inc., Chicago, IL, USA). First, we analyzed the equality of variance and the distribution of the data. We assessed the normality of the data using the Kolmogorov –Smirnov test and the visual inspection of histograms. The normal distribution of quantitative data were presented as mean ± standard deviation (SD), and the abnormal distribution of quantitative data were presented as median (quartile range)., The independent-samples t-test was used for normally distributed continuous variables. The chi-square test for the fourfold table was used for categorical data (when the theoretical frequency was <5, the continuous correction method was adopted and when the theoretical frequency was <1, the exact probability method was adopted). The theoretical frequency of data in the EQ-5D table is too small, thus, the chi-square test of the fourfold table for analysis was used by combining the “Moderate problems” and “Severe problems” data. The correlation between serum S100B levels and GCS scores was analyzed using Spearman’s rank-coefficient test. Mann-Whitney test was used to test for abnormal distribution of continuous variables. Kaplan-Meier survival curves were created and compared using the log-rank test. Receiver operating characteristic (ROC) curves were used to evaluate the ability of S100B1 and S100B3 to diagnose SAE and predict 28-day mortality. The correlation between variables was analyzed by the Pearson’s linear regression test for normal distribution data or Spearman’s rank-coefficient test for non-normal distribution data. All statistical tests were two-tailed, and p < 0.05 was considered to be statistically significant.

## Results

**Figure 1:**
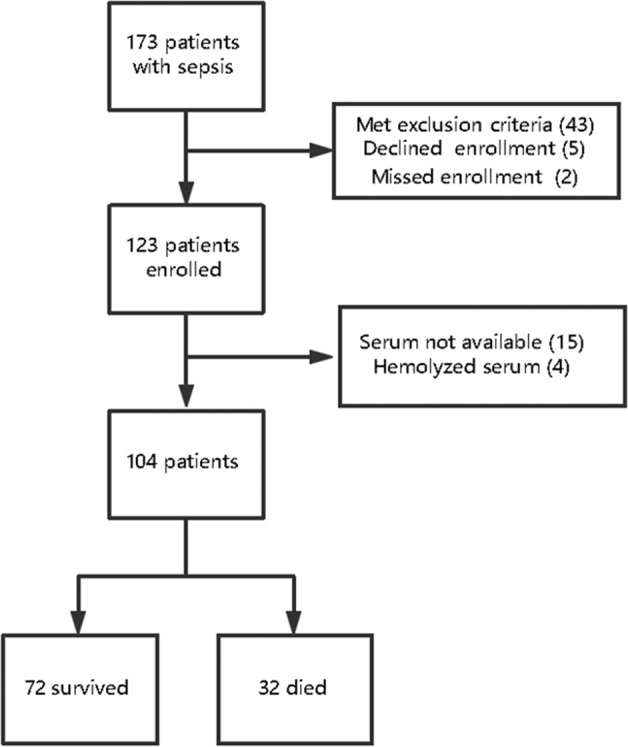
Patient cohorts.

A total of 173 patients admitted to the ICU who presented sepsis were screened for the study. Of these patients, 69 met the exclusion criteria; thus, they were not included in the study. A total of 104 patients were included in the study population, with 59 in the SAE group and 45 in the non-SAE group (Fig. 1).

### Baseline features and biochemical indicators of patients in the SAE and non-SAE groups

There were no significant differences in mean age and gender composition between the SAE and non-SAE groups. Disease severity was significantly higher in the SAE group compared with the non-SAE group (APACHE II score was 20 [17–5] vs. 17 [9–16], p < 0.001; and SOFA score was 11 [9–14] vs. 5 [4–9], p < 0.001). Likewise, the length of ICU stay (7 [4–11] vs. 4 [3–9], p = 0.005), the 28-day mortality (45.76% vs. 11.11%, p < 0.001), and 180-day mortality (54.24% vs. 24.67%, p = 0.005) were significantly higher in the SAE group compared with those in the non-SAE group respectively. Additionally, patients in the SAE group had a higher incidence of shock and gram-negative bacteria infection.

Some biochemical indicators showed statistical differences between the two groups. White blood cell (WBC) count, blood platelet (PLT) count, and procalcitonin (PCT) were higher in the SAE group compared with those in the non-SAE group on day 3. Serum lactate (Lac) and Interleukin-6 (IL-6) were higher on days 1 and 3, and creatinine (Cr) was higher on day 1 in the SAE group compared with those in the non-SAE group. However, the oxygenation index (PaO2/FiO2) was significantly lower on days 1 and 3 in the SAE group compared with those in the non-SAE group. No significant differences in other biochemical indicators were observed (Table 1).

**Table 1:** Baseline Features and Biochemical Indicators of the patients Between SAE group and Non-SAE group.

Parameters	All Patients	SAE group	No-SAE group	p values
	(n = 104)	(n = 59)	(n = 45)	
Age, years (mean ± SD)	56 ± 14	54 ± 15	58 ± 14	0.164
Gender, n (Male/Female)	70/34	38/21	32/13	0.691
APACHE II scores	17(11–22)	21(17–25)	17(9–16)	**<0.001**
Max SOFA scores	9(6–13)	11(9–14)	5(4–9)	**<0.001**
Shock, yes (%)	55(55.77)	44(74.58)	11(24.44)	**<0.001**
GCS scores	14(12–15)	13(12–13)	15(15–15)	**<0.001**
LOS ICU, days	6(3–9)	7(4–11)	4(3–9)	**0.005**
28-day mortality, n (%)	32(31.77)	27(45.76)	5(11.11)	**<0.001**
180-day mortality, n (%)	44(42.31)	32(54.24)	12(26.67)	**0.005**
Source of infection				
*Lung*	14(13.46)	11(18.64)	3(6.67)	0.076
*Abdominal cavity*	72(69.23)	40(67.80)	32(71.11)	0.717
*Urinary tract*	14(13.46)	7(11.86)	7(15.56)	0.863
*Others*	4(3.85)	1(1.69)	3(6.67)	0.183
Bacteriological categories				
*Gram-negative bacteria*	53(50.96)	36(61.12)	17(37.78)	**0.019**
*Gram-positive bacteria*	30(28.85)	16(27.12)	14(31.11)	0.656
*Fungal*	13(12.50)	9(15.25)	4(8.89)	0.331
*Mixed infection*	22(21.15)	15(25.42)	7(15.56)	0.222
Blood culture positive, n(%)	23(22.12)	13(22.03)	10(22.22)	0.982
Number of comorbidities ≥1	48(46.15)	27(45.76)	21(46.67)	0.927
WBC ×10^9^/L	9.2(4.9–15.7)	8.0(3.6–14.9)	10.6(7.1–17.5)	**0.032**
PLT ×10^9^/L	100(50–172)	94(42–149)	115(70–213)	**0.034**
MPV, fl	9.65(8.59–10.95)	9.8(8.58–10.88)	9.42(8.58–11.1)	0.682
S100B on day 1, μg/L	0.217 (0.115–0.430)	0.291 (0.174–0.478)	0.157 (0.09–0.218)	**<0.001**
S100B on day 3, μg/L	0.140 (0.082–0.276)	0.226 (0.129–0.447)	0.089 (0.053–0.136)	**<0.001**
Procalcitonin on day 1, ng/ml	24.6(5.9–65.8)	31.3(10.1–69.0)	16.0(3.9–62.6)	0.064
Procalcitonin on day 3, ng/ml	15.9(3.9–43.1)	23.8(7.3–72.4)	7.52.4–32.6)	**0.001**
Serum Lactate on day 1, mmol/L	2.35(1.23–3.58)	2.9(2.0–4.5)	1.5(1.0–2.5)	**<0.001**
Serum Lactate on day 3, mmol/L	1.15(0.90–2.08)	1.7(1.0–2.4)	0.9(0.6–1.1)	**<0.001**
Creatinine on day 1, µmol/L	127.7 (87.1–206.5)	137.0 (102.1–210.7)	105.5 (77.7–213.2)	**0.042**
Creatinine on day 3, µmol/L	98.3 (72.1–145.4)	107.9 (80.9–146.2)	78.7 (66.6–146.0)	0.056
PH on day 1	7.34 ± 0.11	7.33 ± 0.12	7.35 ± 0.09	0.368
PH on day 3	7.42 ± 0.07	7.41 ± 0.07	7.42 ± 0.06	0.473
PaO2/FiO2 on day 1	262 ± 134	235 ± 133	297 ± 129	**0.019**
PaO2/FiO2 on day 3	262 ± 98	239 ± 97	291 ± 92	**0.007**
Interleukin-6 on day 1, pg/L	226.2 (82.5–1385.8)	534.7 (93.3–5000)	138.2 (68.1–387.3)	**0.001**
Interleukin-6 on day 3, pg/L	129.5 (50.6–338.7)	220.6 (83.6–1002.0)	59.6 (41.3–165.4)	**<0.001**

APACHE, Acute Physiology and Chronic Health Evaluation score; Max SOFA score, maximum Sequential Organ Failure Assessment score evaluated at the fourth day of inclusion; GCS, Glasgow Coma Scale; ICU, intensive care unit; LOS, length of stay; MPV, mean platelet volume. Results are expressed as mean ± SD or median (interquartile range), chi-square test, independent two-samples t-test and Mann-Whitney U test were used for comparison between SAE group and No-SAE group.

### Serum S100B levels for the diagnosis of SAE and prediction of 180-day mortality

**Figure 2:**
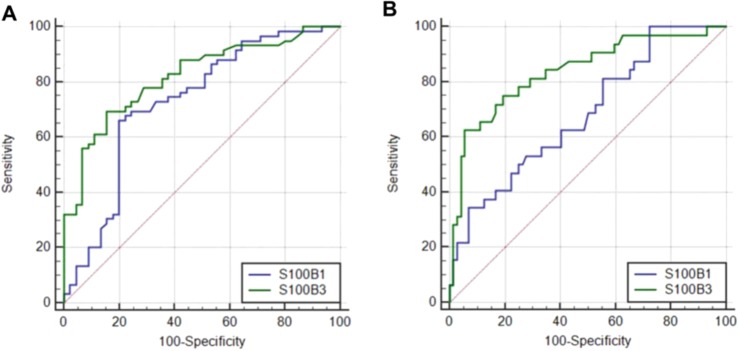
Receiver operating characteristic curve(ROC) of S100B1 (blue line), S100B3(green line) to diagnose SAE (**A**) and to predict the 180-day mortality (**B**). AUCs: S100B1 (**A**) 0.728 (95% CI 0.632–0.810); S100B3 (**A**) 0.819 (95% CI 0.732–0.888); S100B1 (**B**) 0.611 (95% CI 0.510–0.705); S100B3 (**B**) 0.731 (95% CI 0.625–0.813).

The effectiveness of the serum S100B level on day 1 and on day 3 in the diagnosis of SAE was analyzed using the ROC curve. The cut-off value of S100B for day 1 was 0.226 μg/L and for day 3 was 0.144 μg/L. The sensitivity was 66.1% and 69.49% on days 1 and 3, respectively. The specificity was 80.0% and 84.44% for days 1 and 3, respectively. The area under the curve (AUC) was 0.728 (95% CI 0.632–0.811) on day 1 and 0.819 (95% CI 0.732–0.888) on day 3. The positive likelihood ratio (+LR) was 3.31 and 4.47 on days 1 and 3, respectively. The negative likelihood ratio (-LR) was 0.42 and 0.36 on days 1 and 3, respectively (Fig. 2A).

The effectiveness of serum S100B levels on days 1 and 3 in the prediction of the 180-day mortality was analyzed using the ROC curve. The cut-off value of the serum S100B level on day 1 was 0.529 μg/L, with 84.44% specificity and 69.49% sensitivity. Additionally, the AUC was 0.611 (95% CI 0.510–0.705), the +LR was 5.91, and the -LR was 0.74 on day 1. On day 3, the cut-off value of S100B levels was 0.266 μg/L, with 93.33% specificity and 50.0% sensitivity. The AUC was 0.731 (95% CI 0.625–0.813), the +LR was 7.50, and the -LR was 0.54 on day 3 (Fig. 2B).

### Serum S100B levels on day 3 were closely associated with SAE and poor prognosis

The serum S100B levels on day 3 (S100B3) (p = 0.001), but not the serum S100B levels on day 1 (S100B1) (p = 0.927), independently correlated with SAE after adjusting for disease severity and sex (Table 2). There was a stronger correlation between GCS scores and S100B3 compared with that between GCS scores and S100B1 (−0.604 vs. −0.364, respectively).

**Table 2:** Logistic Regression analysis the S100B1 and S100B3 for SAE.

Parameters	OR	95% CI		p	OR	95% CI		p
Gender	4.240	0.577	31.165	0.156	0.881	0.300	2.585	**0.818**
APACHE II - GCS scores	1.189	1.086	1.302	**<0.001**	1.151	1.047	1.265	0.004
S100B1	1.045	0.406	2.690	0.927	**—**	**—**	**—**	**—**
S100B3	**—**	**—**	**—**	**—**	2.263 × 10^4^	66.545	7.695 × 10^5^	**0.001**

APACHE II - GCS scores represent the remaining score in the APACHE II scores minus the GCS scores.

**Figure 3:**
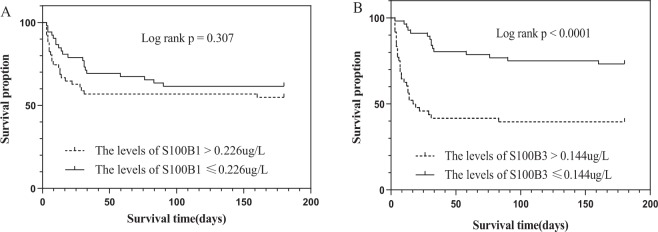
Kaplan-Meier survival analysis according to the cut-off values of S100B levels on day 1 (**A**) and S100B levels on day 3 (**B**) for diagnose of SAE.

Patients were divided into two groups, based on the cut-off values of S100B for the diagnosis of SAE. Patients with S100B levels higher than 0.226 μg/L on day 1 had a similar probability of survival at day 180 compared with patients who had S100B levels lower than 0.226 μg/L (Fig. 3A, p = 0.307). Patients with S100B levels higher than 0.144 μg/L on day 3 had a lower probability of survival at day 180 (Fig. 3B, p < 0.001).

In the SAE group, serum S100B levels of survivors were not statistically different from those of non-survivors on day 1 (p = 0.142); however, the serum S100B levels of survivors were lower than those of non-survivors on day 3 (p < 0.001). In the non-SAE group, there was no statistically significant difference in S100B levels between survivors and non-survivors on days 1 (p = 0.847) and 3 (p = 0.847) (Table 3).

**Table 3:** S100B1 and S100B3 between Survivors and Non-survivors in SAE and No-SAE groups.

Parameters	SAE group		p^b^ values	No-SAE group		p^b^ values
	Survivors^a^	No-survivors		Survivors^a^	No-survivors	
S100B1	0.255 (0.146–0.347)	0.340 (0.179–0.641)	0.142	0.157 (0.085–0.218)	0.142 (0.097–0.222)	0.847
S100B3	0.157 (0.101–0.232)	0.365 (0.177–0.629)	<0.001*	0.089 (0.053–0.136)	0.084 (0.048–0.156)	0.847

^a^Survive at 180 days. Data are given as median (inter-quartile range), ^b^Mann-Whitney U test.

### Dynamic changes in serum S100B levels were associated with SAE and poor prognosis

**Figure 4:**
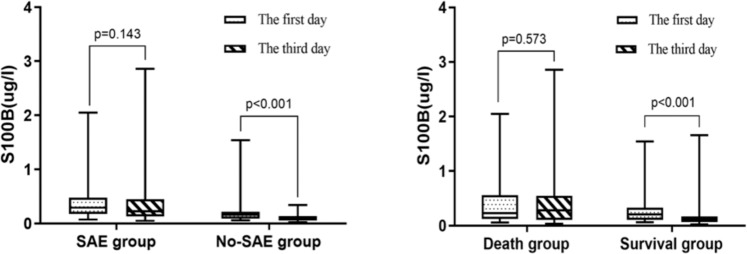
Box-plot representation of S100B levels. Data are shown as box plot with medians (lines inside boxes), 25th and 75th quartiles (limits of boxes); whiskers indicate the range. S100B levels at SAE and No-SAE group (left) and at Survivors and Non-survivors group (right) on day 1 and day 3.

In the SAE group, there was no statistically significant difference between the serum S100B levels on days 1 and 3 (p = 0.143). Similar results were observed in the non-survival group (p = 0.573). By contrast, the serum S100B levels on day 3 were significantly lower than the serum S100B levels on day 1 in the non-SAE group and the survival group (p < 0.001) (Fig. 4).

There were more patients with encephalopathy and a higher 28-day or 180-day mortality in the ΔS100B + group compared with those in the ΔS100B- group (Table 4). APACHE II scores, SOFA scores, GCS scores, Lac on day 1, Lac on day 3, and IL-6 on day 3 were statistically different between the ΔS100B + and ΔS100B- groups (p < 0.05). Multiple logistic regression analysis showed that SAE and serum IL-6 levels on day 3 independently correlated with the dynamic increase in the S100B level (Table 5).

**Table 4:** Incidence of SAE, 28-day and 180 day mortality in ΔS100B + and ΔS100B- group.

Parameters	ΔS100B + group (n = 31)	ΔS100B- group (n = 73)	p^a^ values
SAE, n (%)	27(87.10)	32(43.84)	<0.001*
28-day mortality, n (%)	20(64.52)	12(16.44)	<0.001*
180-day mortality, n (%)	23(74.19)	21(28.77)	<0.001*

ΔS100B means the value of S100B on day 3 minus the value on day 1, ΔS100B + means greater than 0, ΔS100B- means less than or equal to 0.

**Table 5:** Multiple Logistic Regression analysis for the dynamic increase of S100B.

Parameters	OR	95% CI		p	OR	95% CI		p
SAE (Encephalopathy)	4.023	1.124	14.399	**0.032**	4.011	1.105	14.560	**0.035**
APACHE II - GCS scores	1.051	0.953	1.159	0.322	1.043	0.944	1.151	0.407
IL-6 on day 3	1.001	1.000	1.001	**0.017**	1.001	1.000	1.001	**0.037**
Lac on day 1	1.046	0.858	1.27	0.656	**—**	**—**	**—**	**—**
Lac on day 3	**—**	**—**	**—**	**—**	1.273	0.935	1.733	0.126

APACHE II - GCS scores represent the remaining score in the APACHE II score minus the GCS related score.

### Association between serum S100B levels and long-term quality of life

The cutoff point for follow-up was 180 days. Successful follow-up was conducted with 60 patients. The quality of life for sepsis patients was poor, especially in usual activities and physical pain. There was no difference in the EQ-5D scores between the SAE group and the non-SAE group. Patients were grouped into two based on the cut-off values of S100B level for the diagnosis of SAE. However, there was no difference in the EQ-5D scores between the two groups on days 1 and 3.

## Discussion

The main finding of this study was that the dynamic changes in serum S100B levels from day 1 to day 3 were associated with brain dysfunction and fatal prognosis in patients with sepsis. The results obtained in this study support the notion that the dynamic detection of serum S100B levels is a better and more effective method to monitor brain injury in sepsis.

Our previous work published in 2014 on the evaluation of the role of serum S100B and NSE in the diagnosis of SAE demonstrated that serum S100B was a better biomarker than NSE^26^. The sample size and the severity of illness (APACHE II scores) in the present study were similar to those in the previous study. However, this study observed a higher incidence of SAE. The morbidity rate observed in this study was similar to the results of a recent French prospective large multicenter study^30^. The introduction of CAM-ICU criteria enabled an increased detection in the number of patients who presented with delirium, which could explain why a higher incidence of SAE was observed in the present study. The lower mortality rate in the current study, particularly in the non-SAE group, may be credited to optimized treatment methods. Our earlier study included more patients with lung infection while the present study included more patients with abdominal infection. However, the severity of illness and the incidence of SAE in patients with lung infection were significantly higher in the present study compared with those in the previous study. The higher serum S100B level in this study compared to that in the previous study could be due to the increased age of patients, a higher number of patients with positive blood culture, and a higher lactate level of patients.

Although a study reported that elevated serum S100B levels could not reflect the severity of sepsis encephalopathy, the study was a retrospective analysis, had a small sample size, and excluded patients with septic shock^25^. Another study showed that endotoxin-induced short-term inflammation could not trigger brain damage as manifested by an increase in serum S100B level^31^. It is widely believed that sepsis is not caused by a transient systemic inflammatory response but by an unbalanced host response. A systematic review evaluated the role of S100B in SAE from 2001 to 2010^32^. Four studies have shown that elevated serum S100B levels are associated with brain injury and increased mortality in sepsis^20,33–35^. Since 2010, some studies have also verified that elevated serum S100B levels are associated with sepsis brain damage^10,26,28,36^.

Although an increasing number of studies have confirmed that serum S100B levels are elevated in sepsis brain damage, these studies have not been able to accurately assess sepsis brain injury within the initial 24 hours from septic onset. A kinetic model for the dynamic change in serum S100B levels after primary traumatic brain injury (TBI)^37^ showed that even small differences in the sampling time can lead to significant changes in S100B levels during the initial days after injury. A study showed that the peak level of serum S100B was observed at approximately 27 hrs after TBI^16^. Another study also demonstrated that a secondary peak of serum S100B beyond 48 hrs after TBI was strongly correlated with later pathological findings in CT scan and MRI^38^. Since sepsis-associated brain injury is considered a secondary brain injury, the peak time of S100B may be delayed; therefore, we detected serum S100B levels on day 3.

The brain is the primary source of S100B during sepsis^39^. The half-life of S100B is theoretically short; thus, serum S100B levels should decrease quickly when there is no release from an ongoing brain injury. For sepsis patients with sustained brain damage, serum S100B levels may continually increase, especially in patients with severe encephalopathy. In this study, there was no significant difference in serum S100B levels from day 1 to day 3 in the SAE group; however, S100B levels on day 3 were significantly lower than S100B levels on day 1 in the non-SAE group. The efficiency of S100B levels on day 3 for the diagnosis of SAE and the prediction of mortality was superior to S100B levels on day 1. Moreover, our study showed that S100B levels on day 3, but not S100B levels on day 1, were an independent correlation factor for SAE. In congruence with our results, a study showed that there was no significant difference in serum S100B levels in sepsis between the brain dysfunction group and the non-brain dysfunction group on day 1, but there were significant differences on days 2, 3, and 4^28^. Overall, the results of this analysis showed that the serum S100B levels on day 1 were imprecise as biomarkers of sepsis brain injury.

Our study showed that the correlation between GCS scores and S100B levels on day 3 was better than that on day 1 (r = −0.604 vs. −0.364). Although a study reported that elevated S100B levels could not reflect the severity of brain injury during sepsis, all patients with a GCS score ≤ 8, except one patient, in their study had elevated serum S100B levels^25^. In another study, S100B levels in patients with lower GCS scores were higher^20^. The GCS score was used to assess the level of consciousness, but not the content of consciousness. Patients with delirium may, therefore, show no significant change in their GCS scores. Thus, the GCS score may not fully reflect the severity of SAE. Meanwhile, S100B could not reflect all the pathological changes associated with brain injuries in sepsis. Therefore, further investigation is needed to evaluate the severity of SAE.

A recent large-scale study has shown that the mild alteration of mental status is independently associated with mortality in sepsis^30^. Similar to previous studies^20,26,28,40,41^, our results indicated that patients in the SAE group had higher APACHE II and SOFA scores, as well as higher patient mortality. In line with the diagnosis of SAE, the serum S100B level on day 3 had higher efficacy in predicting the 28-day or 180-day mortality compared with the serum S100B level on day 1. However, the main improvement was in specificity, not sensitivity. Brain dysfunction may aggravate organ dysfunction and increase mortality by causing disorders of the cardiovascular system and the neuroendocrine system^8,42^, brain dysfunction does not necessarily lead to death. The prediction of mortality in sepsis is complicated. Clinically, the APACHE scores, the SOFA scores, and others are usually used to improve the sensitivity and specificity of the mortality prediction by integrating multiple indicators.

In sepsis, systemic insults such as impaired cerebral perfusion and micro-circulation, severe hypoxemia, and inflammatory cytokines may contribute to brain injury and peripheral tissue damage. These factors could lead to the first S100B release peak. Transient elevation of S100B could accompany an increase in BBB permeability without brain injury^43,44^ or result from surgical tissue injury or renal failure. In the present study, there was a weak correlation between serum S100B levels and serum creatinine levels on day 1, but there was no correlation between serum S100B levels and creatinine levels on day 3. A study indicated that S100B from an injured skeletal muscle would fully normalize within 20 hrs, and the increased serum S100B levels can be used to reliably evaluate brain injury without continued muscle injury after 24 hrs^45^. Therefore, S100B levels on day 1 may not be a good indicator of brain damage and the dynamic detection of S100B can exclude relevant influencing factors. In our study, the incidence of encephalopathy was approximately 90% in the ΔS100 + group and in 4 patients without encephalopathy. There were 3 patients whose S100B levels were below 0.115 µg/L on day 1, while the increase in the amplitude of S100B was less than 0.01 ug/L. Furthermore, the 180-day mortality rate was 74%, which was significantly higher than that of sepsis and the SAE group. By contrast, the incidence of encephalopathy was only 43.78% in the ΔS100- group, and the mortality rate was also significantly lower. Just as lactate clearance can better reflect perfusion in septic shock patients compared to lactate concentration at a single time point, the dynamic changes in S100B can better reflect brain injury. Through regression analysis, we found that SAE was the main factor for the dynamic increase in S100B.

The EQ-5D scale is a global and multidimensional scoring system for the evaluation of the quality of life^46^. The results of the present study show that there was a significant decrease in the quality of life of the sepsis survivors, especially in physical pain and daily activities. A recent study showed that the increased of serum S100B and E-selectin levels could predict long-term cognitive impairment in critically ill patients^47^. However, in the present study, the EQ-5D scale was no difference in the two groups (which were grouped by the cutoff values of S100B1 or S100B3 and determined by ROC for the diagnosis of SAE). The small sample size may account for this result.

The present study had some limitations. First, due to practical challenges and clinical safety, we could not obtain enough imaging data. Studies have shown that SAE has no specific structural imaging changes, except for some small lesions. These lesions cannot be detected effectively by CT scan; thus, MRI is required for their detection. However, MRI examination requires a long inspection time and cannot be performed at the bedside of the patient. Additionally, many types of medical equipments cannot be brought into the MRI examination room; thus, it is challenging to perform an MRI examination in the early critical state of SAE. Second, for economic reasons and exploratory research, we could only detect S100B levels on days 1 and 3 but not daily. Therefore, some vital information could not be obtained in the present study. Future studies should be designed to include multiple monitoring times and detailed kinetics of S100B. Third, although short-acting sedatives were used for sedation, 24 hrs may not be long enough for drug elimination in some patients and may influence the observation of the conscious state. Fourth, this is a single-center study and the sample size is relatively small; thus, the reproducibility of our results may be affected. Large multi-center studies are necessary to confirm our findings in the future.

## Conclusion

In summary, elevated serum S100B levels on day 3 and the dynamic changes in serum S100B levels from day 3 to 1 were closely associated with brain dysfunction and mortality in sepsis patients. In the future, monitoring the dynamic changes in serum S100B levels could be a better way of observing the occurrence and progression of sepsis-associated encephalopathy.

## Data Availability

The data used to support the findings of this study are available from the corresponding author upon request.
